# The MotoNet: A 3 Tesla MRI-Conditional EEG Net with Embedded Motion Sensors

**DOI:** 10.3390/s23073539

**Published:** 2023-03-28

**Authors:** Joshua Levitt, André van der Kouwe, Hongbae Jeong, Laura D. Lewis, Giorgio Bonmassar

**Affiliations:** 1 Department of Biomedical Engineering, Boston University, Boston, MA 02215, USA; 2 Athinoula A. Martinos Center for Biomedical Engineering, Massachusetts General Hospital, Charlestown, MA 02129, USA

**Keywords:** ballistocardiogram, Kalman adaptive noise cancellation, position estimation, EEG/fMRI

## Abstract

We introduce a new electroencephalogram (EEG) net, which will allow clinicians to monitor EEG while tracking head motion. Motion during MRI limits patient scans, especially of children with epilepsy. EEG is also severely affected by motion-induced noise, predominantly ballistocardiogram (BCG) noise due to the heartbeat. Methods: The MotoNet was built using polymer thick film (PTF) EEG leads and motion sensors on opposite sides in the same flex circuit. EEG/motion measurements were made with a standard commercial EEG acquisition system in a 3 Tesla (T) MRI. A Kalman filtering-based BCG correction tool was used to clean the EEG in healthy volunteers. Results: MRI safety studies in 3 T confirmed the maximum heating below 1 °C. Using an MRI sequence with spatial localization gradients only, the position of the head was linearly correlated with the average motion sensor output. Kalman filtering was shown to reduce the BCG noise and recover artifact-clean EEG. Conclusions: The MotoNet is an innovative EEG net design that co-locates 32 EEG electrodes with 32 motion sensors to improve both EEG and MRI signal quality. In combination with custom gradients, the position of the net can, in principle, be determined. In addition, the motion sensors can help reduce BCG noise.

## 1. Introduction

Multimodal imaging technologies have advanced substantially over the past decade, offering the prospect for high spatiotemporal resolution brain imaging in patients. However, motion artifacts remain a significant limitation for simultaneous electroencephalography (EEG) and magnetic resonance imaging (MRI). Motion artifacts can significantly decrease the quality and diagnostic value of the acquired images [1], making it impossible for radiologists to extract clinically relevant information [2]. Although all subjects are prone to motion artifacts, this problem is particularly severe in pediatric populations due to their difficulty in following instructions [3], especially at specific developmental ages [4]. MRI scans tend to have long acquisition times (often over 30 min) compared to other imaging modalities, such as CTs and plain X-ray films, which instead last minutes or seconds [5]. As a result, children usually need to be sedated to acquire clinically articulate MR images. This anesthesia care introduces additional risks that can vary by the type of sedation, from mild headaches to the suppression of respiratory drive, and sedative drugs may affect brain development in young children [6]. For this reason, the patient needs to be closely monitored by an expert team (including an anesthesiologist or pediatric intensivist) during MRI scans, which significantly increases the cost of the procedure [2]. In addition, the increased need for staff with the required expertise can heavily complicate scheduling, leading to significant delays and compromising timely diagnosis and treatment in many centers. While methods exist to limit and correct the effects of motion in MRI, they have important limitations, such as the inability to account for through-plane motion, and they may require increased scan time, the use of expensive tracking equipment, or sensors attached to the child’s face [4,7]. Developing techniques for efficient correction of motion artifacts in acquired images would increase their diagnostic value and significantly decrease the cost of diagnostic studies by reducing the total scan time and the need for anesthesia.

In addition to interfering with MRI image quality, motion also introduces substantial artifacts into EEG signals. These motion artifacts have largely precluded the use of EEG-MRI in many patient populations. At Boston Children’s Hospital (BCH), for example, it is a routine clinical practice to perform clinical MRIs in neonates undergoing therapeutic hypothermia with MRI-conditional EEG leads in place during the scan. However, the EEG data itself is rarely recorded in the MRI due in part to its contamination with motion artifacts, as young children are highly prone to movements such as startles, limb movements, and head jerks. Adult patients with respiratory difficulties can also exhibit a pathophysiologic response that involves increased breathing work [8], which can also introduce motion artifacts. Each of these types of motion will induce noise in the EEG because of Faraday’s law, as conductors moving inside magnetic fields generate eddy currents. A particularly problematic artifact is the ballistocardiogram, caused by the pulsatile motion of the heart, which must be removed for EEG data to be interpretable [9].

Multiple techniques have been developed to address the important problem of reducing motion artifacts in MRI. If no motion-tracking information is available, images can be reconstructed offline using classical methods, such as autofocus optimization, to minimize an image quality metric such as image entropy [10], thus reducing artifacts. Machine learning has also been used to translate motion-corrupted images into (apparently) “motion-free” images, based on prior knowledge [11]. Motion correction can improve substantially when the motion is explicitly measured, using the physics of the image acquisition process and the mathematics of image reconstruction to correct the errors due to motion. With these measurements, motion can be corrected retrospectively, in post-processing, or prospectively, in real-time [12]. Prospective correction may be more successful in cases where information is missing because of the motion, such as when the person moves outside of the field of view [13]. Motion can be measured with external camera-based systems such as a markerless head tracking system [14] and systems with markers attached to the face [15]. Such hardware is accurate and fast, but expensive. An inexpensive software-only option is to use embedded MR-based navigators, such as FID navigators [16], fat navigators [17], or volumetric/PROMO navigators [18,19]. Still, these must be tailored for the specific imaging sequence. Navigators and camera-based systems are commonly used to track and correct motion in real-time [15], but these require sequence changes or a clear line-of-sight to the patient in the head coil. Small pick-up coils rigidly attached to the patient’s head have been used previously for motion tracking [20], and others have shown that motion tracking is feasible during simultaneous EEG-fMRI using standard MRI conditional EEG amplifiers [21] or an EEG cap with additional loops and high-speed signal sampling [22]. However, these methods sense motion in the same EEG channel, further complicating the BCG artifact rejection.

In addition to reducing image artifacts, motion tracking can also be used to improve the quality of EEG recordings. EEG acquired inside the MRI scanner is contaminated by ballistocardiogram (BCG) noise caused by cardiac pulsatile motion [23]. This noise must be removed in post-processing for the EEG data to be usable. An effective approach for artifact removal uses a reference layer to insulate a subset of EEG channels from the scalp, providing direct measurements of motion-related noise which can then be used for artifact removal. However, these reference-layer methods have not been widely adopted, as the layer is not integrated into the EEG nets, and it can be challenging to build [24].

Here, we introduce a novel net optimized for EEG-fMRI with motion sensors, the MotoNet. The key innovation is that each electrode is embedded with a motion sensor in a thin circuit forming a 32-channel EEG/motion sensing net, enabling: (1) the detection of fine electrode motion for ballistocardiogram noise estimation for EEG signal cleaning and (2) the detection of bulk motion using 32 electrodes for robust position estimation of the entire head. In addition, we present an adaptive cancellation filter to reduce the ballistocardiogram noise and a method to estimate head position in an MRI, both specifically developed for this new net. The motion sensors in the MotoNet provide noise measurements for subsequent BCG noise reduction using Kalman filtering (see [25] and MotoNet examples below).

The MotoNet design employs 32 loops/sensors embedded alongside 32 EEG electrodes. A key novel aspect is the arrangement of a motion-detecting loop, parallel and in close proximity to each EEG electrode. Since the voltage-induced flux in each sensor depends on the position and orientation of the sensor in the gradient field [26], the combination of rigidly related sensors allows for the tracking of bulkhead motion. This measurement is more precise than what is possible with EEG electrodes alone, as they are less focal, as each electrode records gradient artifact signals from the entire volume conductor of the head. Furthermore, BCG noise is different in every electrode [27], as they can move independently from each other, with some channels experiencing very low motion (i.e., occipital). Therefore, the 32 motion sensors in the MotoNet enabled us to develop electrode-specific BCG reduction for improved artifact removal.

The MotoNet is designed to effectively suppress RF-induced heating to be safely used in MRI and is compatible with a commercial EEG amplifier system, making it well suited to patient use. In addition, it is manufactured using cost-effective materials and processes, enabling wide application in clinical studies. The MotoNet could enable the EEG-fMRI measurements of pediatric subjects, for both basic neuroscience and clinical applications, as well as facilitate neuroimaging in patients with epilepsy, by mitigating motion-induced noise in both EEG and MRI.

## 2. Materials and Methods

**Figure 1:**
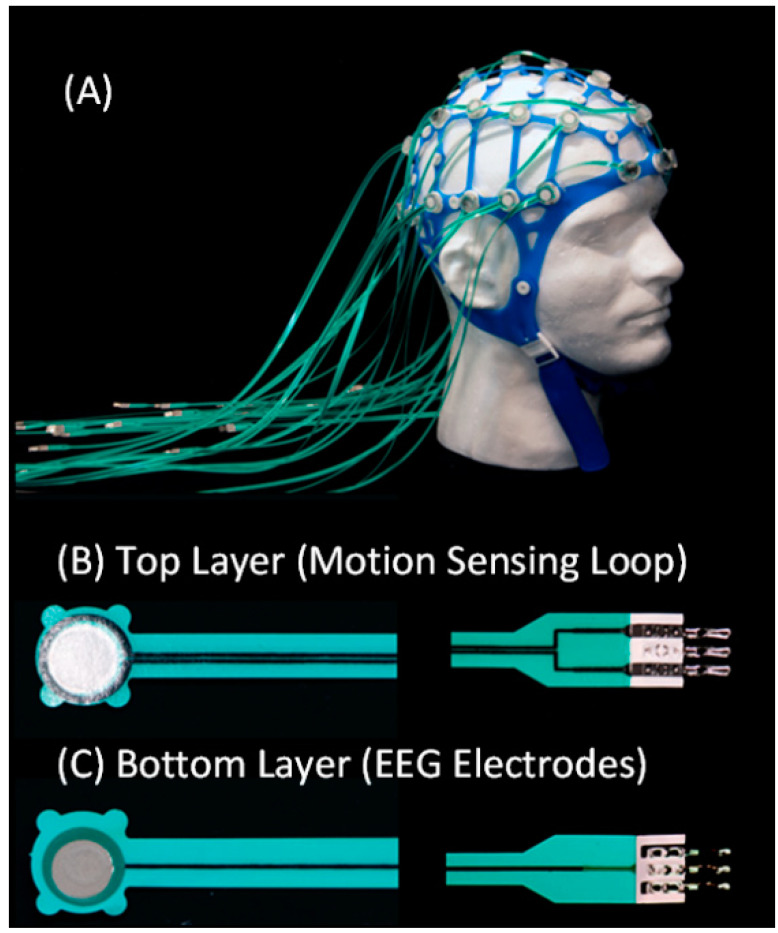
The 32-channels/motion sensors net. Image of the actual MotoNet (**A**). Image of the double sided PTF leads with a PTF coil on one side (**B**) and a traditional EEG electrode (**C**) on the opposite side.

**Figure 2:**
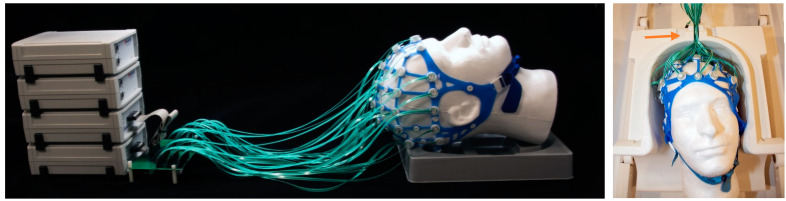
MotoNet setup. The MotoNet is connected to two commercial EEG amplifiers through a custom-made interface (**left**). The traces have been designed to slide into the Siemens 64-channel head/neck coil (orange arrow on the **right**).

- *Fabrication:* High-resistance polymer thick film (PTF) technology (Figure 1) was used to fabricate electrode pads and leads. Conductive inks (Engineered Conductive Materials, Delaware, OH, USA) were screen-printed onto Melinex (DuPont Teijin Films U.S. Limited Partnership, Chester, VA, USA) substrate, with EEG electrodes on one side and a loop on the opposite side. A dielectric ink (ECM) was used to coat the leads and loops for electrical and environmental insulation. The MotoNet circuits were fit to a commercial elastomer structure (Figure 1 and Figure S6). An adapter (Figure 2 and Figure S1) was built to interface the MotoNet to two EEG amplifiers (Brain Products GmbH, Gilching, Germany), with the EEG channels and the motion sensors routed to different amplifiers in order to have separate grounds and references.
- *Safety*: The Radiofrequency (RF) safety of the MotoNet was tested in a 3 T Prisma (Siemens Healthineers, Erlangen, Germany) MRI using an adult head-sized agar phantom. The dielectric properties of the phantom were selected to be similar to the adult brain properties at 128 MHz [28,29], i.e., σ = 0.52 S/m, ε_r_ = 65.4. For temperature measurements, 8-channel fiber optic probes (OSENSA Innovations Corp., Coquitlam, BC, Canada) were positioned at distributed locations across the MotoNet, including three hot spots estimated from the thermal simulation [30]. Thermal paste was used to keep the fiber optic probes in contact with the surface of the agar phantom and EEG electrodes, to assess RF-induced heating. A high-power turbo spin-echo sequence (21 slices, 0.9 × 0.9 × 5.0 mm voxels, TR/TE = 7600/86 ms, FA = 120°, 20 averages) was used to deliver 100% SAR for 30 min (SAR_head_: 3.07 W/kg, 10 g SAR_torso_ local: 9.60 W/kg) to reach the maximum power deposition within the allowable RF safety limit in a clinical scan.
- *Movement Estimation:* We assume that the subject’s head wearing the MotoNet is an ideal sphere centered at the origin with surface points P(*x*,*y*,z) satisfying the equation:
  (1) $$x^{2}+y^{2}+z^{2}=r^{2}$$

We will consider only the following rigid transformations: rotation and translation. The new position of the sphere after rigid transformation can be written as:

(2) $$\left(\begin{array}{c} x^{\prime }\\ y^{\prime }\\ \begin{array}{c} z^{\prime }\\ 1 \end{array} \end{array}\right)=M\left(\begin{array}{c} x\\ y\\ \begin{array}{c} z\\ 1 \end{array} \end{array}\right)=R\left(\begin{array}{c} x\\ y\\ \begin{array}{c} z\\ 1 \end{array} \end{array}\right)+\left(\begin{array}{c} x_{0}\\ y_{0}\\ \begin{array}{c} z_{0}\\ 1 \end{array} \end{array}\right)$$

where **M** is the transformation matrix, and$\;x^{\prime },\;y^{\prime },\;$and $z^{\prime }$ are the coordinates of the point P($x^{\prime },\;y^{\prime },\;z^{\prime }$) after rigid transformation, $x_{0}$, $y_{0}$, $z_{0}$ are in the shift components (in meters or m), and R is the rotation matrix around the three axis $R=R_{x}R_{y}R_{z}$ as follows:

(3) $$R_{x}=\left[\begin{array}{cccc} 1 & 0 & 0 & 0\\ 0 & cos\left(\alpha_{x}\right) & -sin\left(\alpha_{x}\right) & 0\\ 0 & sin\left(\alpha_{x}\right) & cos\left(\alpha_{x}\right) & 0\\ 0 & 0 & 0 & 1 \end{array}\right]R_{y}=\left[\begin{array}{cccc} cos\left(\alpha_{y}\right) & 0 & sin\left(\alpha_{y}\right) & 0\\ 0 & 1 & 0 & 0\\ -sin\left(\alpha_{y}\right) & 0 & cos\left(\alpha_{y}\right) & 0\\ 0 & 0 & 0 & 1 \end{array}\right]R_{z}=\left[\begin{array}{cccc} cos\left(\alpha_{z}\right) & -sin\left(\alpha_{z}\right) & 0 & 0\\ sin\left(\alpha_{z}\right) & cos\left(\alpha_{z}\right) & 0 & 0\\ 0 & 0 & 1 & 0\\ 0 & 0 & 0 & 1 \end{array}\right]$$

Or more compactly, it can be written as:

(4) $$R_{x}=\left[\begin{array}{cccc} 1 & 0 & 0 & 0\\ 0 & c_{x} & -s_{x} & 0\\ 0 & s_{x} & c_{x} & 0\\ 0 & 0 & 0 & 1 \end{array}\right]R_{y}=\left[\begin{array}{cccc} c_{y} & 0 & s_{y} & 0\\ 0 & 1 & 0 & 0\\ -s_{y} & 0 & c_{y} & 0\\ 0 & 0 & 0 & 1 \end{array}\right]R_{z}=\left[\begin{array}{cccc} c_{z} & -s_{z} & 0 & 0\\ s_{z} & c_{z} & 0 & 0\\ 0 & 0 & 1 & 0\\ 0 & 0 & 0 & 1 \end{array}\right]$$

Thus, the rotation matrix can be written compactly as:

(5) $$R=\left[\begin{array}{cccc} c_{y}c_{z} & -c_{y}S_{z} & s_{y} & 0\\ c_{x}S_{z}+s_{x}S_{y}c_{z} & c_{x}c_{z}-s_{x}S_{y}S_{z} & -S_{x}c_{y} & 0\\ S_{x}S_{z}-c_{x}S_{y}c_{z} & c_{x}S_{y}S_{z}+s_{x}c_{z} & c_{x}c_{y} & 0\\ 0 & 0 & 0 & 1 \end{array}\right]$$

The rigid transformation is completely determined by estimating the six parameters: $x_{0},\;y_{0},\;z_{0}{,\;\alpha }_{x},\;\alpha_{y},\;\alpha_{z}$, which will be estimated by using MotoNet’s motion sensors. The last three unknowns can be expressed in terms of the matrix R:

(6) $$\left\{\begin{array}{c} \alpha_{x}=atan2\left(-\frac{R_{2,3}}{c_{y}},\frac{R_{3,3}}{c_{y}}\right)\\ \alpha_{y}=sin^{-1}\left(R_{1,3}\right)\\ \alpha_{z}=atan2\left(\frac{R_{1,2}}{c_{y}},-\frac{R_{1,1}}{c_{y}}\right) \end{array}\right.$$

Equation (2) can be written more explicitly as:

(7) $$\left\{\begin{array}{c} x^{\prime }=xc_{y}c_{z}-yc_{y}s_{z}+zs_{y}+x_{0}\\ y^{\prime }=x\left(c_{x}s_{z}+s_{x}s_{y}c_{z}\;\right)+y\left(c_{x}c_{z}-s_{x}s_{y}s_{z}\right)-zs_{x}c_{y}+y_{0}\\ z^{\prime }=x\left(s_{x}s_{z}-c_{x}s_{y}c_{z}\right)+y\left(c_{x}s_{y}s_{z}+s_{x}c_{z}\right)+zc_{x}c_{y}+z_{0} \end{array}\right.$$

The voltage $v_{i}\;\left(t\right)$ induced in a loop i = 1:32 depends on the orientation of the loop with respect to the changing magnetic flux:

(8) $$v_{i}\;\left(t\right)=a_{xi}\;\left(t\right)\;\frac{\partial B_{x}\;\left(t\right)}{\partial t}+a_{yi}\;\left(t\right)\frac{\partial B_{y}\;\left(t\right)}{\partial t}+a_{zi}\;\left(t\right)\frac{\partial z_{x}\;\left(t\right)}{\partial t}$$

where the orientation is encoded in the areas *a_xi_*, *a_yi_*, and *a_zi_* of the sensor loop projected onto the planes perpendicular to the normal in the x, y, and z directions, respectively. The location of the coil is encoded by the gradients, which are designed to generate a field with a z-component that varies linearly with the distance from the gradient isocenter. However, Maxwell’s equations also predict orthogonal field components [31]:

(9) $$\left(\begin{array}{c} B_{x}\left(t\right)\\ B_{y}\left(t\right)\\ B_{z}\left(t\right) \end{array}\right)=\left(\begin{array}{ccc} -\frac{G_{z}\left(t\right)}{2} & 0 & G_{x}\left(t\right)\\ 0 & -\frac{G_{z}\left(t\right)}{2} & G_{y}\left(t\right)\\ G_{x}\left(t\right) & G_{y}\left(t\right) & G_{z}\left(t\right) \end{array}\right)\left(\begin{array}{c} x\\ y\\ z \end{array}\right)$$

Since the motion sensor detects the time-varying component of the field, the magnet’s **B**_0_ static field is irrelevant. Furthermore, we design the time-varying gradient waveforms to vary with a sinusoidal function with the same amplitude G (mT/m/ms) that switches ON and OFF alternatively according to $u_{x}\left(t\right)$, $u_{y}\left(t\right),$ and $u_{z}\left(t\right)\;$(i.e., periodic boxcar functions), as follows [32]:

(10) $$\left\{\begin{array}{c} G_{x}\left(t\right)=Gsin\left(\omega t\right)u_{x}\left(t\right)\\ G_{y}\left(t\right)=Gsin\left(\omega t\right)u_{y}\left(t\right)\\ G_{z}\left(t\right)=Gsin\left(\omega t\right)u_{z}\left(t\right) \end{array}\right.$$

We averaged the response across the harmonic waveforms, accounting for the sign. This waveform is repeated independently twice on the *x*-axis and once for the y- and z-axes with the same amplitude. We therefore expect the induced voltages *v_xi_*, *v_yi_*, and *v_zi_* to depend on position and orientation before and after motion at an interval Δt. We assume that the motion is small for a small t, or that the loop area does not change with a small motion (for simplicity, we use the equal sign from here forward). For simplicity, we have dropped the temporal component from the notation, and we only consider the amplitude of the harmonic voltages. Thus, the voltages induced in a loop i = 1:32 at a position P(*x_i_*, *y_i_*, *z_i_*) are as follows [31]:

(11) $$\left(\begin{array}{c} v_{xi}\\ v_{yi}\\ v_{zi} \end{array}\right)=A\left(\begin{array}{ccc} a_{zi} & 0 & a_{xi}\\ 0 & a_{zi} & a_{yi}\\ -\frac{a_{xi}}{2} & -\frac{a_{yi}}{2} & a_{zi} \end{array}\right)\left(\begin{array}{c} x_{i}\\ y_{i}\\ z_{i} \end{array}\right)\;$$

where $v_{xi},\;v_{yi},\;\;\mathrm{and}\;v_{zi}$ are the voltages of the i^th^ loop in response to the $G_{x}\left(t\right),\;G_{y}\left(t\right),\;$and $G_{z}\left(t\right)$ gradients in the two different positions. A is the coefficient between the sensor space location and the voltage space. Equation (11) can be written more explicitly as:

(12) $$\left\{\begin{array}{c} v_{xi}=A\left(z_{i}a_{xi}+x_{i}a_{zi}\right)\\ v_{yi}=A\left(z_{i}a_{yi}+y_{i}a_{zi}\right)\\ v_{zi}=A\left(z_{i}a_{zi}-\frac{x_{i}a_{xi}+y_{i}a_{yi}}{2}\right) \end{array}\right.$$

In order to estimate the areas *a_xi_*, *a_yi_*, and *a_zi,_*,we note that for the MotoNet, the area of the electrodes (*r*_e_ = 5 mm) is $C=\pi r_{e}^{2}=78.54\;{\mathrm{mm}}^{2}$. Furthermore, we can write the areas *a_xi_*, *a_yi_*, and *a_zi_* as areas of circles projected into a plane [33]:

(13) $$\left\{\begin{array}{c} a_{xi}=C\;cos\left(\gamma_{xi}\right)\\ a_{yi}=C\;cos\left(\gamma_{yi}\right)\\ a_{zi}=C\;cos\left(\gamma_{zi}\right) \end{array}\right.$$

where $\gamma_{xi}$ is the angle between the *x*-axis and the normal vector to the sphere passing through P_i_($x_{i},y_{i}$, $z_{i}$), and similarly for $\gamma_{yi}\;and\;\gamma_{zi}$. In order to estimate $\gamma_{xi}$_,_ $\gamma_{yi}\;\mathrm{and}\;\gamma_{zi}$, we note that normal $\vec{n}$ in a generic point P_i_($x_{i},y_{i}$, $z_{i}$) of the sphere is given by $\vec{n}$ = ∇f in Equation (1) $f\left(x,y,z\right)=x^{2}\;$+ $y^{2}$+ $z^{2}-r^{2}$:

(14) $$\vec{n}\;\left(P_{i}\right)=\left(\frac{\partial f\left(x,y,z\right)}{\partial x},\frac{\partial f\left(x,y,z\right)}{\partial x},\frac{\partial f\left(x,y,z\right)}{\partial x}\right)=\left(x_{i},y_{i},\;z_{i}\right)=\vec{i}\;x_{i}+\vec{j}\;y_{i}+\vec{k}\;z_{i}$$

where $\vec{i},\;\vec{j}\;\mathrm{and}\;\vec{k}\;$are the standard Cartesian versors. Since the sphere is centered in the origin, the vector normal to its surface is the vector from the origin. Thus:

(15) $$\left\{\begin{array}{c} \gamma_{xi}=acos\frac{\vec{i}\cdot \vec{n}\;\left(P_{i}\right)}{\left\|\vec{n}\;\left(P_{i}\right)\right\|}=acos\frac{x_{i}}{\sqrt{x_{i}^{2}+y_{i}^{2}+z_{i}^{2}}}=acos\frac{x_{i}}{r}\\ \gamma_{yi}=acos\frac{\vec{j}\cdot \vec{n}\;\left(P_{i}\right)}{\left\|\vec{n}\;\left(P_{i}\right)\right\|}=acos\frac{y_{i}}{\sqrt{x_{i}^{2}+y_{i}^{2}+z_{i}^{2}}}=acos\frac{y_{i}}{r}\\ \gamma_{zi}=acos\frac{\vec{k}\cdot \vec{n}\;\left(P_{i}\right)}{\left\|\vec{n}\;\left(P_{i}\right)\right\|}=acos\frac{z_{i}}{\sqrt{x_{i}^{2}+y_{i}^{2}+z_{i}^{2}}}=acos\frac{z_{i}}{r} \end{array}\right.$$

Equation (13) results in:

(16) $$\left\{\begin{array}{c} a_{xi}=\left|\frac{x_{i}C}{r}\right|\\ a_{yi}=\left|\frac{y_{i}C}{r}\right|\\ a_{zi}=\left|\frac{z_{i}C}{r}\right| \end{array}\right.$$

From Equations (12) and (16), we finally obtain:

(17) $$\left\{\begin{array}{c} v_{xi}=A\left(\frac{x_{i}z_{i}C}{r}+\frac{z_{i}x_{i}C}{r}\right)=\frac{2ACx_{i}z_{i}}{r}+v_{x0}\\ v_{yi}=A\left(\frac{z_{i}y_{i}C}{r}+\frac{z_{i}y_{iC}}{r}\right)=\frac{2ACy_{i}z_{i}}{r}+v_{y0}\\ v_{zi}=\frac{AC}{R}\left(z_{i}^{2}-\frac{x_{i}^{2}+y_{i}^{2}}{2}\right)+v_{z0} \end{array}\;\right.\;$$

where $v_{x0},\;v_{y0},\;\;and\;v_{z0}\;$are the boundary field voltages. Equation (17) is intended to be solved in the least square sense, as it is an overdetermined system of equations (i.e., 32 equations with 7 variables).

- *Phantom Motion Tracking Scans*: We placed the head-shaped agar phantom in a 3 T Siemens Prisma scanner and imaged it in the initial position, plus three other positions. The x-, y- and z-gradient responses were measured and ensemble-averaged across epochs (128 repetitions). We translated the phantom, approximately guided by a measured template. The EEG system was set up to record EEG signals at the highest sampling rate available (5000 S/s) with no filter settings beyond those present in the hardware. The *sinusoidal amplitude estimation* was performed as follows: prior to finding the amplitude of each sinusoidal response, each was normalized by subtracting out its mean to remove DC drift. The amplitude of each normalized sinusoidal response was then calculated as the amplitude of the 500 Hz sine wave that minimized the root mean square error (RMSE) between that sine wave and the normalized sinusoidal response.
- *EEG Recordings in a Human Subject.* To test the Kalman noise cancellation capability of the MotoNet, we recorded EEG on a healthy adult subject wearing the MotoNet. The Massachusetts General Hospital Institutional Review Board approved the study procedures, and the subject provided informed consent. The MotoNet EEG signals were measured inside the 3 T scanner while the participant alternated between periods of eyes open and eyes closed states. The PTF leads were passed through the opening at the top of a Siemens 64-channel head/neck coil and connected to a 64-channel EEG amplifier system (BrainProducts GMBH) through a custom-made interface (see Figure 2 or Supplementary Material Figure S1).
- *EEG Kalman Noise Cancellation*: To remove noise, EEG signals recorded inside a 3 T MRI on a human volunteer were Kalman filtered as follows. We modeled the *n*-channel recorded EEG scalar signal $y_{i,t}$ as the sum of a “true” underlying EEG (scalar) signal $s_{i,\;t}$ and $m_{t}^{T}$, or the motion sensor signal vector with size 1 × (*n* + 1) (i.e., a motion sensor for each EEG channel) multiplied by $x_{i,\;t}\;,\;$or the weight vector with size (*n* + 1) × 1 and *t* = 1…*T*:
  (18) $$y_{i,t}=s_{i,\;t}+m_{t}^{T}\cdot x_{i,t}$$
  where *i* and *t* are indices tracking the current EEG channel number (i.e., spatial index) and sample number (i.e., temporal index), respectively; *n* is the number of EEG or motion sensors channels (i.e., a motion sensor for each EEG electrode, plus one additional constant value result in *n* + 1 channels), and ‘·’ is the vector or matrix product. The $x_{i,\;t}$ was initialized prior to beginning the filtering procedure to a vector of zeros. The state $x_{i,\;t}\;$can be found (see Figure S3):
  (19) $$x_{i,\;t}=x_{i,\;t-1}+g_{i,t}\left(y_{i,t}-m_{t}^{T}\cdot x_{i,t}\right)$$
  where $g_{i,t}$ is the following Kalman gain vector with size (*n* + 1) × 1:
  (20) $$g_{i,t}={\hat{P}}_{i,t}\cdot m_{t}{\left(m_{t}^{T}\cdot {\hat{P}}_{i,t}\cdot m_{t}+R\right)}^{-1}\;$$
  where R is the measurement noise covariance scalar, a constant model hyperparameter, in this case, estimated to be 100, and ${\hat{P}}_{i,t}$ is the error covariance matrix at the current step of size (*n* + 1) × (*n* + 1):
  (21) $${\hat{P}}_{i,t}=P_{i,\;t-1}+Q$$
  where ***Q*** is the noise covariance matrix, which is a model hyperparameter and is held constant and is assumed to be an identity matrix **I** of size (*n +* 1) × (*n +* 1) multiplied by a scalar (estimated to be 0.01 in this case), and where $P_{i,\;t-1}$ is the error covariance matrix (at the previous time step), which is initialized prior to each recording to be an identity matrix of size (*n* + 1) × (*n* + 1). Finally, ${\hat{P}}_{i,t}$ and $P_{i,\;t}$ are also related as follows, limiting the unbounded increase in ${\hat{P}}_{i,t}\;$in Equation (21):
  (22) $$P_{i,\;t}=\left(I-g_{i,t}\cdot m_{t}^{T}\right)\cdot {\hat{P}}_{i,t}$$

**Figure 3:**
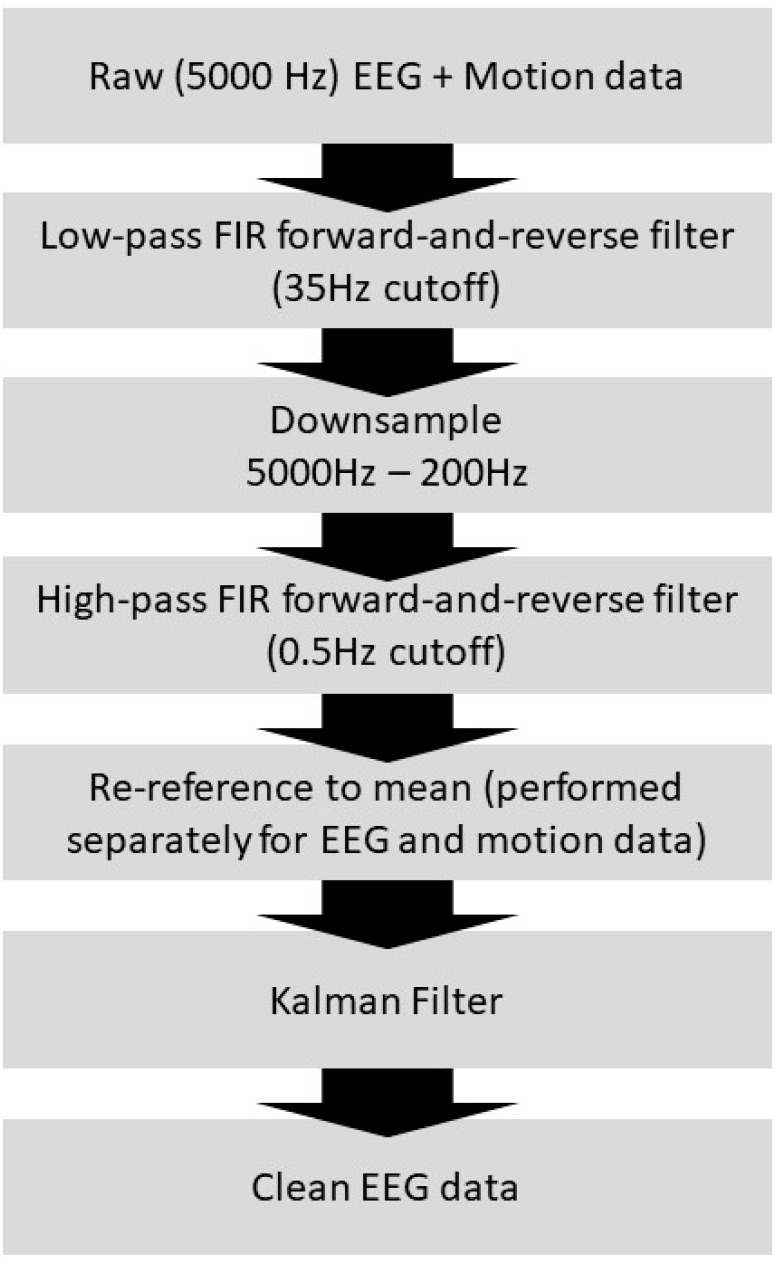
Flowchart for the EEG processing and Kalman filtering.

Custom MATLAB code was used to implement Equations (18)–(22), which is presented in the Supplementary Materials. Prior to noise cancellation, the EEG signals were forward- and reverse low-pass filtered using an FIR filter with a cutoff frequency of 35 Hz and then down-sampled from 5 kHz to 200 Hz (Figure 3). As a comparison point for noise removal, average artifact subtraction (AAS) [34] was applied using the FMRIB toolbox for EEGLAB (Figure 10), as it is one of the most-used BCG correction methods.

To measure the computational complexity and duration of the artifact removal algorithm, we used it to clean segments of an EEG recording of various lengths (50 s, 100 s, 150 s, 200 s, 250 s, and 300 s). For each segment, we applied the algorithm using a subset of either 8, 16, 24, and 32 channels. We repeated this process, varying the length of segments in the same way, with the number of EEG channels fixed at 32, but varying the number of motion channels used as exogenous inputs to be 8, 16, 24, or 32. These experiments were performed to measure computational time and how it varies with respect to recording duration, the number of EEG channels, and the number of motion inputs. All the computational time measurements were repeated 10 times using a single core of a laptop with an Intel i7-8565U processor. An exponential function was fit to computation time (relative to the number of sensors) in MATLAB using the *fminserch* function to find the optimal fit to the ten repeated measurements for each of the six-segment lengths.

## 3. Results

We investigated the MR safety of the MotoNet, its ability to estimate displacement in a phantom, and its utility for EEG noise cancellation in human subjects.

### 3.1. MotoNet Fabrication

When constructing the MotoNet, we fabricated the polymer thick-film traces successfully, with no discards. The electrode traces had a resistance of $\bar{R_{trace}}\;$ = 17.47 ± 0.95 kΩ, and the average resistance of the motion sensor loops was $\bar{R_{trace}}\;$ = 41.61 ± 2.07 kΩ. These results achieved our target resistance of 40–45 kΩ, aiming to match the resistance of a standard EEG electrode.

### 3.2. MRI Safety

**Figure 4:**
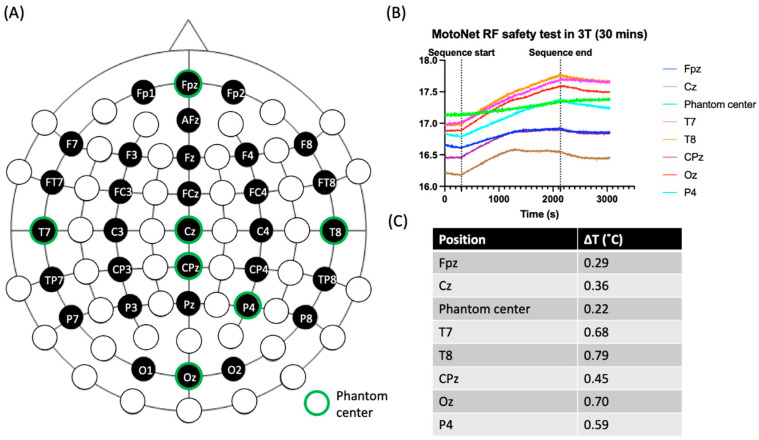
Temperature measurements with head-sized agar phantom. (**A**) The spatial distribution of 32-channel EEG electrodes where temperature was monitored. The eight thermal probes were positioned in the hot-spots identified by simulations, circled in green. (**B**) The temperature elevation in each probe during a high-power turbo spin-echo sequence with the maximum SAR allowed in a clinical scan for 30 min using a birdcage body transmit coil; (**C**) table of the total change in temperature (ΔT) during the scan for each measurement location. The temperature increase was less than 1 °C, well within the safety limits.

To test for MRI safety and ensure no excessive heating with the MotoNet, we performed a 30 min scan delivering 100% SAR to the phantom. Across all thermal probes, the maximum temperature increase during the scan was 0.79 °C. This result is well within FDA limits, demonstrating that the device does not require additional scan restrictions to reduce RF-induced heating, as these heating levels were appropriate for scans lasting 1 h in normal operating mode, without pauses for cooling time [35] (Figure 4).

### 3.3. Displacement Estimation in Phantoms

**Figure 5:**
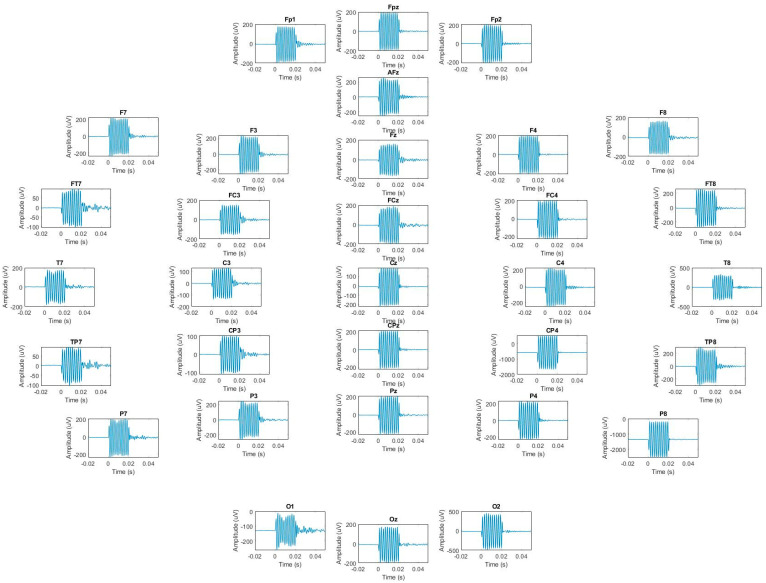
Example of motion sensor signal outputs for the x-axis gradient.

**Figure 6:**
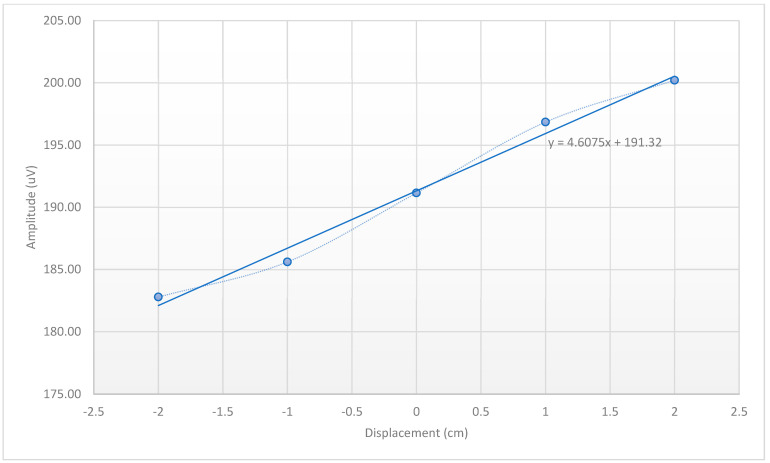
Relationship between the MotoNet motion sensors’ average amplitude (Figure S5) and the mean *x*-axis position, corresponding to 9.552 µV/cm. The circles with a dotted line indicate measured data; the solid line indicates the best-fit line.

Given the safe level of heating in the MotoNet, we next investigated whether it could be used for motion tracking in MRI. We examined whether the motion sensor signals could enable inference of total displacement in the phantom scan. The equation for estimating displacement from the motion sensor measurements is expressed in Equation (17), and we illustrate the calculation here. We limited our analysis to *x*-axis translations for this initial proof of concept study. In the case of only *x*-axis motion, there are only three variables ($v_{x0},\;x\;\mathrm{and}\;A)$ to be estimated in Equation (17). Figure 5 shows the motion sensor signals recorded from the MotoNet using the commercial EEG acquisition system, while we applied our custom sequence. The custom MRI sequence consisted of a sinusoidal gradient with a frequency of 500 Hz and duration of 20 ms (10 cycles), repeated every 200 ms, first on the *x*-axis, then again on the *x*-axis (for test-retest and sync), then on the *y*-axis, and finally, on the *z*-axis before repeating the series of four waveforms. The motion sensors picked up the derivatives of gradient signals generated by the custom sequence, and we considered only the gradients in the x-direction, since the motion was purely along the *x*-axis (i.e., with no *z*-axis motion, $v_{xi}$ varies linearly with the *x*-axis position; see Equation (11)). Figure 6 shows the linear relationship between the average voltages over all the motion sensors and the *x*-axis displacement (see Figure S5 for channel-by-channel data). The data suggest that the sensitivity of the sensors for our sequence is 9.552 × 10^−4^ V/m. The best-fit line relating the average *x*-axis displacement (mm) to motion sensor output ($v_{x}\;\mathrm{in}\;$µV) was:

(23) $$x=\frac{\langle v_{x}\rangle }{k}-41.5\;\mathrm{mm}\;$$

where k = 4.6 µV/mm. The three estimated variables in  Equation (17) were: x, the x-component of the center of the phantom’s head, which can be estimated from Equation (23); $v_{x0}=191.3$ V; and the constant A:

(24) $$A=1.4\bar{6}\;\langle z\rangle =4.4\;V/m^{3}$$

where 〈z〉 was the average z position of all the 32 electrodes on the phantom’s head, or 29.96 mm.

### 3.4. BCG Adaptive Noise Cancellation

**Figure 7:**
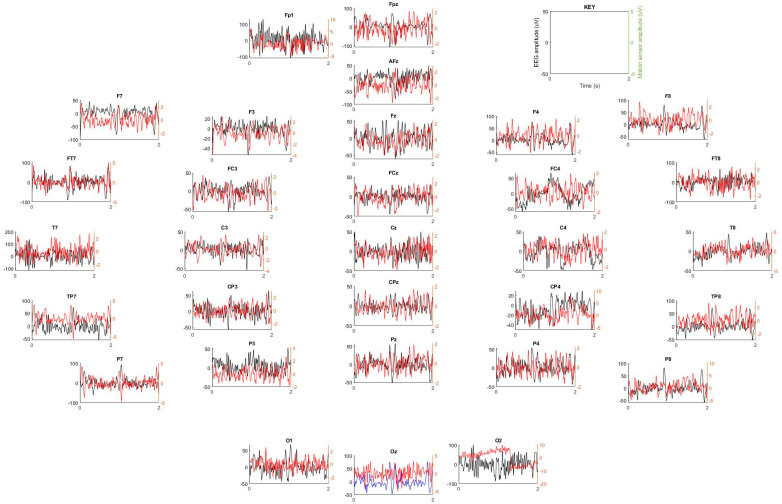
Kalman filter input signals. Each EEG signal (e.g., Oz in blue) corrupted by BCG is cleaned by subtracting a weighted sum of all the motion sensor signals (i.e., in red). The algorithm is then repeated for all EEG signals (i.e., in black).

**Figure 8:**
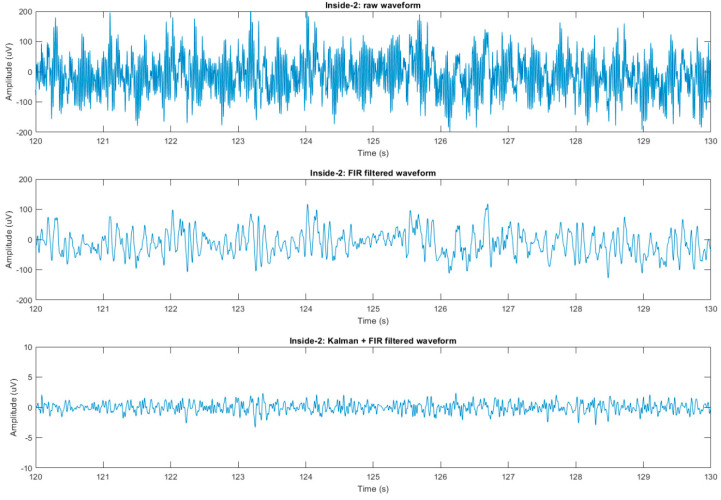
Example of signal cleaning in a single EEG channel. Raw EEG recording inside a 3 T MRI, without scanning (**top**); EEG after low pass filtering (**middle**); and cleaned EEG data after Kalman filtering, using the motion sensor signals (**bottom**).

**Figure 9:**
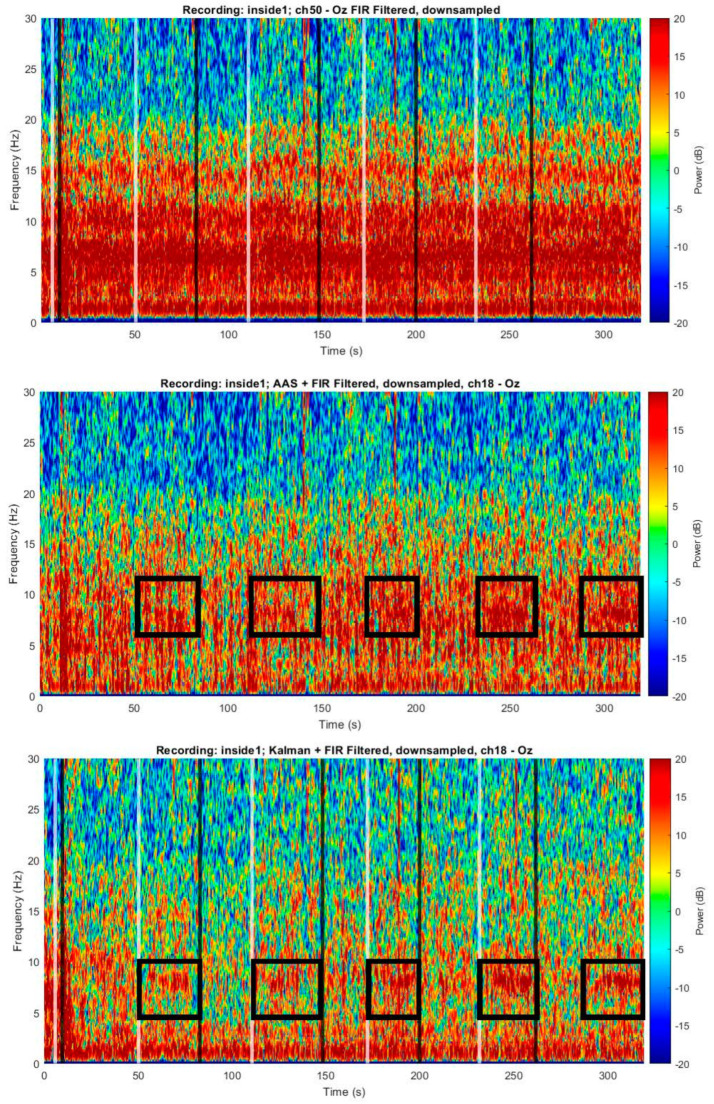
Example of a single EEG channel with adaptive BCG artifact cancellation. Spectrogram of an EEG recording at 3 T with the MotoNet after FIR filtering (**top**) and after Kalman filtering (**bottom**). After cleaning, ~10 Hz alpha rhythms are detected during the eyes-closed periods (black boxes in bottom panel). White vertical lines indicate instructions to close eyes; black vertical lines indicate eye opening.

**Figure 10:**
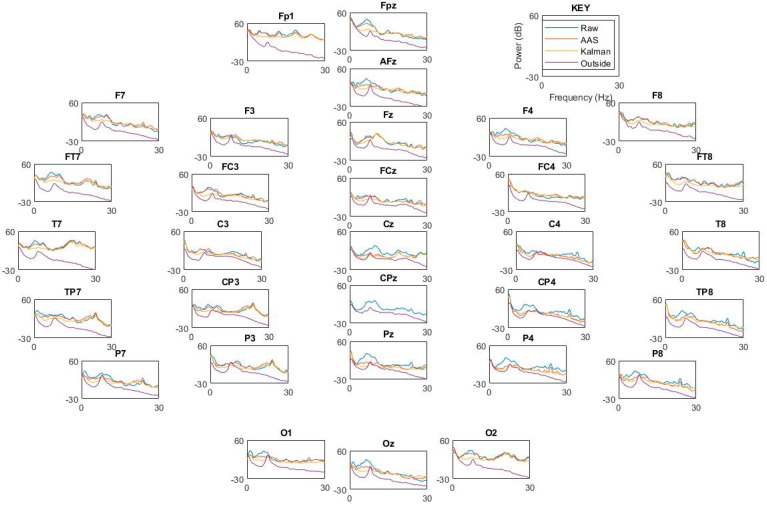
Power spectra per electrode across cleaning methods: unfiltered/raw, AAS [34], Kalman filter, and clean signals acquired outside the MRI scanner field.

We next investigated whether these motion sensor signals could enable the removal of motion-related artifacts from the EEG signal in a human subject. Figure 7 shows an example of the motion sensor signals, which served as input signals for the Kalman filter, in which all motion sensor signals were weighted (Figure S3) and summed to clean each EEG channel. Each motion sensor had a very different signal, demonstrating the importance of having a motion sensor in each channel, as this enabled highly local measurements of the motion at each point in space. Figure 8 illustrates each filtering step for a single channel. We next investigated how this algorithm performed by examining the EEG recorded with eyes open or closed. Figure 9 shows two spectrograms of EEG recordings from an occipital channel collected with the MotoNet inside a 3 Tesla field after FIR filtering (top) and after Kalman filtering using the motion sensor signals (bottom). Strong alpha power (~10 Hz) was seen in the EEG multi-taper spectrogram when the subject closed his/her eyes, as expected. Figure 10 shows the example power spectra of unfiltered/raw EEG, and EEG after cleaning with AAS [34], with Kalman filtering, and EEG recorded outside-the-MRI scanner field, in all 32 channels. The Kalman filter achieved the best adaptive noise cancellation when all motion sensor channels were included (Figure S2), and the minimum RMS value of 1.4 µV for each sensor (Figure S4) was significantly greater than the amplifier’s resolution (i.e., 0.5. µV for the BrainAmp MR plus).

We next measured the processing time for several variations of the EEG cleaning algorithm to estimate the computational complexity of the adaptive noise cancellation procedure. We varied the segment length (i.e., *T*), the number of EEG channels (i.e., $n_{EEG})\;$and the number of motion sensors $\left(n_{motion}\right)$. We found that computation time scaled linearly with both segment length *T* and the number of EEG channels $n_{EEG}$, but exponentially with the number of exogenous inputs $n_{motion}$. The best-fit exponent for the computation time with respect to $n_{motion}$ was 5.5. This suggests that the complexity of this algorithm is approximately $O\left(T*n_{EEG}*n_{motion}^{5.5}\right)$. For the maximum number of EEG channels (i.e., 32), the maximum segment size (300 s), and the maximum number of motion channel inputs (i.e., 32), the mean computation time was 72 s. The computation time with respect to T, $n_{EEG}$, and $n_{motion}$ is summarized in Figure S7.

## 4. Discussion

### 4.1. Safety

The use of electrical devices inside the MRI scanner requires careful attention to potential safety risks from heating. Conductive EEG leads in the presence of a radio-frequency (RF) field generates induced currents [36] and potential increases in RF power absorbed in the human head, quantified as the Specific Absorption Rate (SAR) [37]. For relatively high-power RF sources, such as MRI RF coils, such interactions pose serious thermal-related safety risks in regards to tissue heating and burns [38,39,40,41,42,43]. Our approach uses PTF technology to reduce this risk. The increased lead resistivity provided by PTF/thin-film technology allows for safe and high-quality recordings at fields up to 7 T in adults [44]. Our recent simulation study on EEG safety in infants and toddlers [45] showed a similar finding concerning the low risk of RF heating when using conductive EEG traces in 3 T MRI: thin-film-based resistive traces, with similar resistivity to the one used in the MotoNet, reduced the risk of RF heating as per the Medical Devices for Safety in the MRI Environment guideline of the FDA [35]. PTF technology thus provides a promising approach for safely recording EEG in the MR environment.

### 4.2. Displacement Estimation in Phantoms

We found that our approach enabled the tracking of displacement, as the motion sensor signals were correlated with the position. Motion tracking in an MRI system can be accomplished using small coils attached to the head [31,46], but this requires special hardware for acquisition. Standard MRI conditional EEG amplifiers [47] have been successfully employed to estimate the position of the subject’s head based on the EEG signal. However, this method suffers from many EEG-related artifacts (i.e., eye blinking, muscular twitches, etc.), and it requires filtering to extract the motion component, which is time intensive. Our new MotoNet adopts both strategies: (1) it includes small coils, and (2) it allows the use of MRI conditional EEG amplifiers. This is accomplished by printing the motion sensor coils on the Melinex substrate on the opposite side of each electrode, allowing the subject’s head motion to be measured independently from the EEG and its artifacts, since the motion sensors are electrically isolated from the subject’s head. Furthermore, our design included 32 motion sensors, enabling a least-squares solution of the head motion to an overdetermined system, since in Equation (17), we solve 32 equations with only 7 unknowns (i.e., $x,\;y,\;z,\;v_{x0}{}_{i},v_{y0},v_{z0},\;A$). Using dedicated motion sensors can provide more precise information as compared to inferring motion information from EEG signals, since EEG is less focal and records signal from the entire volume conductor of the head.

An underlying assumption in our approach is that the induced changes in the motion sensor loop signals are linearly related to the subject’s position. We took the average of all the sinusoidal amplitudes from all 32 motion sensors to estimate the overall *x*-axis position of the head phantom, as there was no motion in the y- and z-axes. In general, this does not hold, as in Equation (11), gradients and positions occur in a multivariate relationship. In addition, we developed a rigid motion algorithm to specifically take advantage of the new sensors, which has the benefit of not requiring a subject–specific calibration [22]. Our method does assume that movements are small; however, this assumption is not significantly restrictive in practice, since one can analyze smaller time differences if needed to ensure that this condition holds. Furthermore, movements in the MRI are greatly constrained, limiting the magnitude of the overall displacements. Finally, an additional advantage of the proposed method is the low computational complexity. The optimization required only a few seconds in MATLAB, which was sufficient to allow for the real-time implementation of motion correction in the scanner.

### 4.3. MotoNet Design

The MotoNet design is well suited to future development for clinical use. Previous work from our group [44,48] showed the advantage of PTF over commercially available copper wires in terms of MRI image quality, **B_1_** field homogeneity, and fMRI signal-to-noise ratio. Having 32 EEG and 32 motion sensors requires 64 channels, and this need for an increased channel count may have previously been considered to be a limitation. However, modern commercially available amplification systems can easily allow the acquisition of as many as 256 channels, so the need to double the number of recordings can be easily accommodated with the current hardware. Furthermore, the impedance of the PTF-based loops was chosen to match the normal EEG impedances so that they would not interfere with a high-density amplifier [49]. In this study, the testing was performed using an off-the-shelf EEG amplifier to acquire signals, making this technology potentially available to a wider public. However, a complete development of the MotoNet, enabling it to be used for motion correction directly on the MRI images, will require future work.

### 4.4. BCG Adaptive Noise Cancellation

In addition to motion estimation for MRI, the MotoNet provides a promising approach for improving the quality of EEG recorded inside the scanner. In simultaneous EEG-fMRI, the EEG recordings are severely contaminated by ballistocardiogram (BCG) artifacts caused by cardiac-generated pulsations of the brain and head, which induces noise currents. A unique feature of the MotoNet is that every EEG channel has a paired motion sensor channel, allowing spatially matched measurements of the motion. The BCG artifact from a neighboring position to each EEG channel can then be used to increase the precision of the artifact removal. Our results show the proof of concept that these motion signals can help reduce EEG artifacts acquired in the MRI scanner, as we found that the output of the motion sensors was strong enough to produce a reliable signal for Kalman filtering for artifact reduction (Figure S4).

Our group first proposed a BCG adaptive noise reduction algorithm [25] based on the Kalman filter algorithm, with external measurements of BCG noise, originally using EOG sensors [50]. Intuitively, the adaptive algorithm makes use of any correlation between the motion signal *m*(*t*) and the observed signal *y*(*t*) to estimate the FIR kernel *w*(*t*) and remove the noise signal *n*(*t*). Since the true underlying EEG signal *s*(*t*) is mostly uncorrelated with the motion signal *m*(*t*), the adaptive algorithm should not affect it, and on average, as a result $\hat{s}\left(t\right)\;$of the noise, the cancellation process will be the true underlying EEG. However, in this work, we present a steerable spatial filter, rather than an FIR filter, that adaptively weighs all the motion sensor signals $m_{t}^{T}$ optimally or *steers* the weighted sum $m_{t}^{T}\cdot x_{i,t}$ in the direction of minimal BCG noise. This type of spatial Kalman adaptive noise cancellation has been successfully applied to EEG/fMRI [51].

### 4.5. Limitations

A limitation of this study is that for proof of concept, we only studied translation along the *x*-axis. In this setting, the results corresponded well with our theoretical formulas. We believe that the generalization to the other two remaining axes is straightforward, as demonstrated by Laustsen et al. [22], which can be explored in future work. Because we used conventional EEG acquisition equipment with hardware lowpass filters, we were limited to relatively low-frequency stimulation gradients, which added time to the imaging sequence. However, this is a limitation of the acquisition equipment, not the MotoNet design itself.

A second limitation was that the resistance of the traces increased over time, as is common with PTF. However, this issue did not affect the overall quality of the motion sensor recordings, except for eight manually rejected channels in the final averaging in Equation (23), for which the sinusoidal amplitude estimation algorithm produced nonlinear or incorrect results (Figure S5).

The magnetic fluxes through the traces were considered spurious fluxes, which could, in principle, have affected the measurements. The spurious fluxes may have primarily affected the position estimation, rather than the BCG motion sensing, as the traces were only marginally subject to motion in the eyes open/closed experiments. However, the motion sensors were designed to have only the smallest fabricable spacing between adjacent traces (0.01”, see Figure S6). This design could be improved in the future by using a *multilayer approach*, or by stacking traces on top of each other to minimize the spurious magnetic flux. Following a similar multilayer approach, the PCB was designed with a ground layer to minimize spurious fluxes (Figure S1).

We also found that the computational complexity of this algorithm was sufficiently low for use in real-time experiments, as the time required to process a segment of data was lower than the duration of that segment. This result is unsurprising, as an analogous technique was previously used in real-time [51]. However, it is important to note that this processing time depends upon the specific hardware used to perform the computation, and performance will vary across individual implementations.

The computational complexity we observed is approximately in line with the theoretical expectations for Kalman filters. The linear increase in complexity with the number of time points *T* multiplied by the number of channels $n_{EEG}$ is in line with our theoretical expectations as the solution of a problem in the form of n-chains of m-graphs. Slight deviations from these expectations can be explained by the relatively low number of repetitions performed, the coarse sampling in the channel- and time-space, and variation in CPU load from background processes. The exponential increase in complexity with the increasing number of motion channel inputs *n* is also in line with the theoretical expectations for Kalman filters, although the exponent we observed was higher than expected [52].

## 5. Conclusions

We have shown that the MotoNet is a 3 T MRI conditional and imaging-compatible EEG net for cross-modal neural monitoring, enabling movement tracking and ballistocardiogram (BCG) artifact reduction. The proposed MotoNet is an inexpensive, noninvasive EEG net that overcomes current cross-modal safety concerns and motion artifact issues that severely limit the effectiveness of simultaneous EEG/MRI. Furthermore, the motion-sensing technology is lightweight and small, using advanced polymer thick film manufacturing technologies.

The MotoNet thus allows researchers and clinicians to benefit from the high spatial resolution of MRI and the high temporal resolution of EEG, with mitigated motion-induced noise. We presented a spatial adaptive Kalman noise cancellation algorithm for cleaning the EEG, which takes advantage of MotoNet’s many motion sensors. We also showed that we could estimate the head shift in the x-direction by modeling the sensor positions and orientations based on a custom-made MRI sequence, the signals recorded on the motion sensors, and the known geometry of the MotoNet and of a phantom. In future work, we will take advantage of MotoNet’s large number of motion sensors to capture and motion-correct the MRI of a patient’s head in real-time, especially in the case of children.

The MotoNet can thus enable low-cost, high-quality imaging with motion correction. This technology has broad applications for studying brain function in healthy children and in different neonatal and developmental neural pathologies, such as epilepsy.

## Data Availability

The data presented in this study are available on request from the corresponding author.

## Supplementary Materials

The following supporting information can be downloaded at: https://www.mdpi.com/article/10.3390/s23073539/s1, Figure S1: Breakout box schematic. Figure S2: Eyes open/closed mean power difference for different electrode selections. Figure S3: Example of state vectors $x_{i,t}$. Figure S4: Example of energy in a 10 s recording of motion sensors recorded at 3 T in a volunteer using the MotoNet. Figure S5: Channel-by-channel x-axis shift vs. motion sensors’ amplitude when running the custom-made sequence at 3T MRI. Figure S6: The MotoNet CAD design. At the end of the Supplementary Material is the MATLAB code of the Kalman filter. Figure S7: Computational complexity of the adaptive noise removal algorithm.

## References

[1] Jorge J., Grouiller F., Gruetter R., Van Der Zwaag W., Figueiredo P. (2015). Towards high-quality simultaneous EEG-fMRI at 7 T: Detection and reduction of EEG artifacts due to head motion. Neuroimage.

[2] Schulte-Uentrop L., Goepfert M.S. (2010). Anaesthesia or sedation for MRI in children. Curr. Opin. Anaesthesiol..

[3] Mulert C., Lemieux L., Mulert C., Lemieux L. (2023). EEG-fMRI: Physiological Basis, Technique, and Applications.

[4] Kuperman J.M., Brown T.T., Ahmadi M.E., Erhart M.J., White N.S., Roddey J.C., Shankaranarayanan A., Han E.T., Rettmann D., Dale A.M. (2011). Prospective motion correction improves diagnostic utility of pediatric MRI scans. Pediatr. Radiol..

[5] Placidi G. (2012). MRI: Essentials for Innovative Technologies.

[6] FDA (2017). FDA Drug Safety Communication: FDA approves label changes for use of general anesthetic and sedation drugs in young children. Drug Safety and Availability.

[7] Zaitsev M., Maclaren J., Herbst M. (2015). Motion artifacts in MRI: A complex problem with many partial solutions. J. Magn. Reson. Imaging.

[8] Dani C., Reali M., Bertini G., Wiechmann L., Spagnolo A., Tangucci M., Rubaltelli F. (1999). Risk factors for the development of respiratory distress syndrome and transient tachypnoea in newborn infants. Italian Group of Neonatal Pneumology. Eur. Respir. J..

[9] Allen P.J., Josephs O., Turner R. (2000). A method for removing imaging artifact from continuous EEG recorded during functional MRI. Neuroimage.

[10] Atkinson D., Hill D., Stoyle P., Summers P., Keevil S. (1997). Automatic correction of motion artifacts in magnetic resonance images using an entropy focus criterion. IEEE Trans. Med. Imaging.

[11] Küstner T., Armanious K., Yang J., Yang B., Schick F., Gatidis S. (2019). Retrospective correction of motion-affected MR images using deep learning frameworks. Magn. Reson. Med..

[12] Maclaren J., Herbst M., Speck O., Zaitsev M. (2013). Prospective motion correction in brain imaging: A review. Magn. Reson. Med..

[13] Slipsager J.M., Glimberg S.L., Højgaard L., Paulsen R.R., Wighton P., Tisdall M.D., Jaimes C., Gagoski B.A., Grant P.E., van der Kouwe A. (2022). Comparison of prospective and retrospective motion correction in 3D-encoded neuroanatomical MRI. Magn. Reson. Med..

[14] Slipsager J.M., Ellegaard A.H., Glimberg S.L., Paulsen R.R., Tisdall M.D., Wighton P., Van Der Kouwe A., Marner L., Henriksen O.M., Law I. (2019). Markerless motion tracking and correction for PET, MRI, and simultaneous PET/MRI. PLoS ONE.

[15] Zaitsev M., Dold C., Sakas G., Hennig J., Speck O. (2006). Magnetic resonance imaging of freely moving objects: Prospective real-time motion correction using an external optical motion tracking system. Neuroimage.

[16] Kober T., Marques J.P., Gruetter R., Krueger G. (2011). Head motion detection using FID navigators. Magn. Reson. Med..

[17] Gallichan D., Marques J.P., Gruetter R. (2016). Retrospective correction of involuntary microscopic head movement using highly accelerated fat image navigators (3D FatNavs) at 7T. Magn. Reson. Med..

[18] Tisdall M.D., Hess A.T., Reuter M., Meintjes E.M., Fischl B., van der Kouwe A.J.W. (2012). Volumetric navigators for prospective motion correction and selective reacquisition in neuroanatomical MRI. Magn. Reson. Med..

[19] White N., Roddey C., Shankaranarayanan A., Han E., Rettmann D., Santos J., Kuperman J., Dale A. (2010). PROMO: Real-time prospective motion correction in MRI using image-based tracking. Magn. Reson. Med..

[20] Ooi M.B., Aksoy M., Maclaren J., Watkins R.D., Bammer R. (2013). Prospective motion correction using inductively coupled wireless RF coils. Magn. Reson. Med..

[21] Purdon P.L., Millan H., Fuller P.L., Bonmassar G. (2008). An open-source hardware and software system for acquisition and real-time processing of electrophysiology during high field MRI. J. Neurosci. Methods.

[22] Laustsen M., Andersen M., Xue R., Madsen K.H., Hanson L.G. (2022). Tracking of rigid head motion during MRI using an EEG system. Magn. Reson. Med..

[23] Krishnaswamy P., Bonmassar G., Poulsen C., Pierce E.T., Purdon P.L., Brown E.N. (2016). Reference-free removal of EEG-fMRI ballistocardiogram artifacts with harmonic regression. Neuroimage.

[24] Luo Q., Huang X., Glover G.H. (2014). Ballistocardiogram artifact removal with a reference layer and standard EEG cap. J. Neurosci. Methods.

[25] Bonmassar G., Purdon P.L., Jääskeläinen I.P., Chiappa K., Solo V., Brown E.N., Belliveau J.W. (2002). Motion and Ballistocardiogram Artifact Removal for Interleaved Recording of EEG and EPs during MRI. Neuroimage.

[26] van Niekerk A., Meintjes E., van der Kouwe A. (2019). A Wireless Radio Frequency Triggered Acquisition Device (WRAD) for Self-Synchronised Measurements of the Rate of Change of the MRI Gradient Vector Field for Motion Tracking. IEEE Trans. Med. Imaging.

[27] Abreu R., Leite M., Jorge J., Grouiller F., van der Zwaag W., Leal A., Figueiredo P. (2016). Ballistocardiogram artifact correction taking into account physiological signal preservation in simultaneous EEG-fMRI. Neuroimage.

[28] Duan Q., Duyn J.H., Gudino N., de Zwart J.A., van Gelderen P., Sodickson D.K., Brown R. (2014). Characterization of a dielectric phantom for high-field magnetic resonance imaging applications. Med. Phys..

[29] Hasgall P., Di Gennaro F., Baumgartner C., Neufeld E., Lloyd B., Gosselin M., Payne D., Klingenböck A., Kuster N. (2022). IT’IS Database for Thermal and Electromagnetic Parameters of Biological Tissues. Itis.Swiss/Database.

[30] Atefi S.R., Serano P., Poulsen C., Angelone L.M., Bonmassar G. (2018). Numerical and Experimental Analysis of Radiofrequency-Induced Heating Versus Lead Conductivity During EEG-MRI at 3 T. IEEE Trans. Electromagn. Compat..

[31] Laustsen M., Andersen M., Lehmann P.M., Xue R., Madsen K.H., Hanson L.G. Slice-wise motion tracking during simultaneous EEG-fMRI. Proceedings of the Joint Annual Meeting ISMRM-ESMRMB.

[32] Gholipour A., Polak M., van der Kouwe A., Nevo E., Warfield S.K. Motion-robust MRI through real-time motion tracking and retrospective super-resolution volume reconstruction. Proceedings of the 2011 Annual International Conference of the IEEE Engineering in Medicine and Biology Society.

[33] De Cusatis C., Optical Society of America (1997). Handbook of Applied Photometry.

[34] Allen P.J., Polizzi G., Krakow K., Fish D.R., Lemieux L. (1998). Identification of EEG events in the MR scanner: The problem of pulse artifact and a method for its subtraction. Neuroimage.

[35] FDA, Center for Devices and Radiological Health (2021). Testing and Labeling Medical Devices for Safety in the Magnetic Resonance (MR) Environment.

[36] Chou C.K., Bassen H., Osepchuk J., Balzano Q., Petersen R., Meltz M., Cleveland R., Lin J.C., Heynick L. (1996). Radio frequency electromagnetic exposure: Tutorial review on experimental dosimetry. Bioelectromagnetics.

[37] NCRP (1981). Radiofrequency Electromagnetic Fields: Properties, Quantities and Units, Biophysical Interaction, and Measurement.

[38] Ibrahim T.S., Kangarlu A., Chakeress D.W. (2005). Design and performance issues of RF coils utilized in ultra high field MRI: Experimental and numerical evaluations. IEEE Trans. Biomed. Eng..

[39] Collins C.M., Liu W., Wang J., Gruetter R., Vaughan J.T., Ugurbil K., Smith M.B. (2004). Temperature and SAR calculations for a human head within volume and surface coils at 64 and 300 MHz. J. Magn. Reson. Imaging.

[40] Finelli D.A., Rezai A.R., Ruggieri P.M., Tkach J.A., Nyenhuis J.A., Hrdlicka G., Sharan A., Gonzalez-Martinez J., Stypulkowski P.H., Shellock F.G. (2002). MR imaging-related heating of deep brain stimulation electrodes: In Vitro study. AJNR Am. J. Neuroradiol..

[41] Gajsek P., Walters T.J., Hurt W.D., Ziriax J.M., Nelson D.A., Mason P.A. (2002). Empirical validation of SAR values predicted by FDTD modeling. Bioelectromagnetics.

[42] Noth U., Laufs H., Stoermer R., Deichmann R. (2012). Simultaneous electroencephalography-functional MRI at 3 T: An analysis of safety risks imposed by performing anatomical reference scans with the EEG equipment in place. J. Magn. Reson. Imaging.

[43] Shellock F.G. (2003). Magnetic Resonance Procedures: Health Effects and Safety.

[44] Vasios C.E., Angelone L.M., Purdon P.L., Ahveninen J., Belliveau J.W., Bonmassar G. (2006). EEG/(f)MRI measurements at 7 Tesla using a new EEG cap (“InkCap”). Neuroimage.

[45] Jeong H., Ntolkeras G., Grant P.E., Bonmassar G. (2021). Numerical simulation of the radiofrequency safety of 128-channel hd-EEG nets on a 29-month-old whole-body model in a 3 Tesla MRI. IEEE Trans. Electromagn. Compat..

[46] van Niekerk A., van der Kouwe A., Meintjes E. (2019). Toward “plug and play” prospective motion correction for MRI by combining observations of the time varying gradient and static vector fields. Magn. Reson. Med..

[47] Wong C.K., Zotev V., Misaki M., Phillips R., Luo Q., Bodurka J. (2016). Automatic EEG-assisted retrospective motion correction for fMRI (aE-REMCOR). Neuroimage.

[48] Poulsen C., Wakeman D.G., Atefi S.R., Luu P., Konyn A., Bonmassar G. (2017). Polymer thick film technology for improved simultaneous dEEG/MRI recording: Safety and MRI data quality. Magn. Reson. Med..

[49] van der Kouwe A., Jeong H., Yang Z., Straney D., Frost R., Lewis L., Bonmassar G. The MotoNet: An MRI-Compatible EEG Net with Embedded Motion Sensors. Proceedings of the Joint annual Meeting ISMRM-ESMRMB-ISMRT.

[50] Haykin S.S. (2002). Adaptive Filter Theory.

[51] Levitt J., Yang Z., Williams S., Espinosa S., Garcia-Casal A., Lewis L. (2022). EEG-LLAMAS: An open source, low latency, EEG-fMRI neurofeedback platform. bioRxiv.

[52] Thrun S., Burgard W., Fox D. (2005). Probabilistic Robotics.

