# Supplementary material for: The MotoNet: A 3 Tesla MRI-Conditional EEG Net with Embedded Motion Sensors
Source: Sensors (Basel). 2023 Mar 28;23(7):3539. doi: 10.3390/s23073539 (PMC10098760; doi:10.3390/s23073539)
Supplement: Supplementary file 1 [file sensors-23-03539-s001.zip › sensors-2274781-supplementary.pdf]

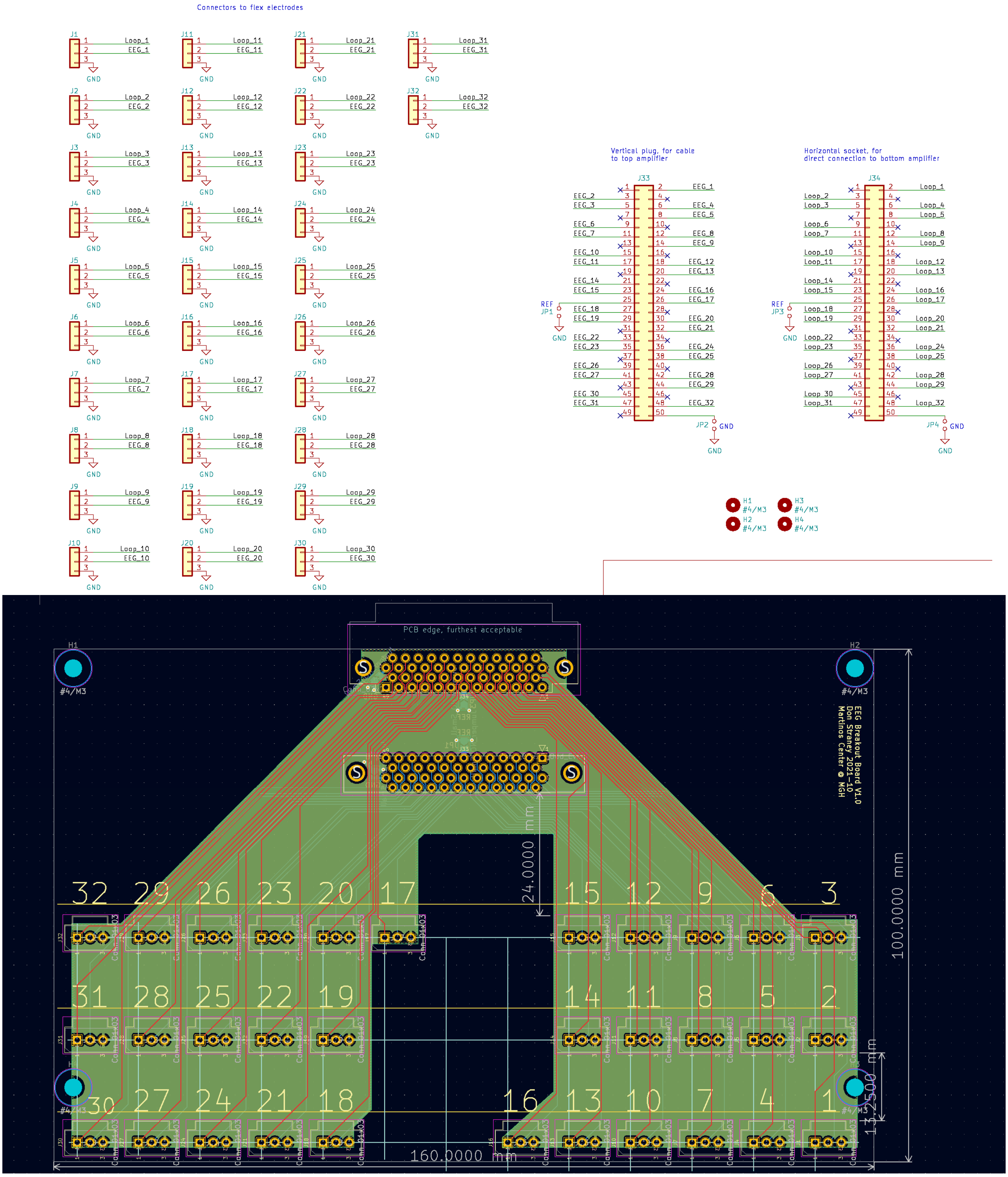

Figure S1: Breakout box (top) schematic and (Bottom) PCB layout. Following a multilayer approach, we implemented a ground layer underneath the 32 EEG lead plugs to reduce the unwanted spurious flux.

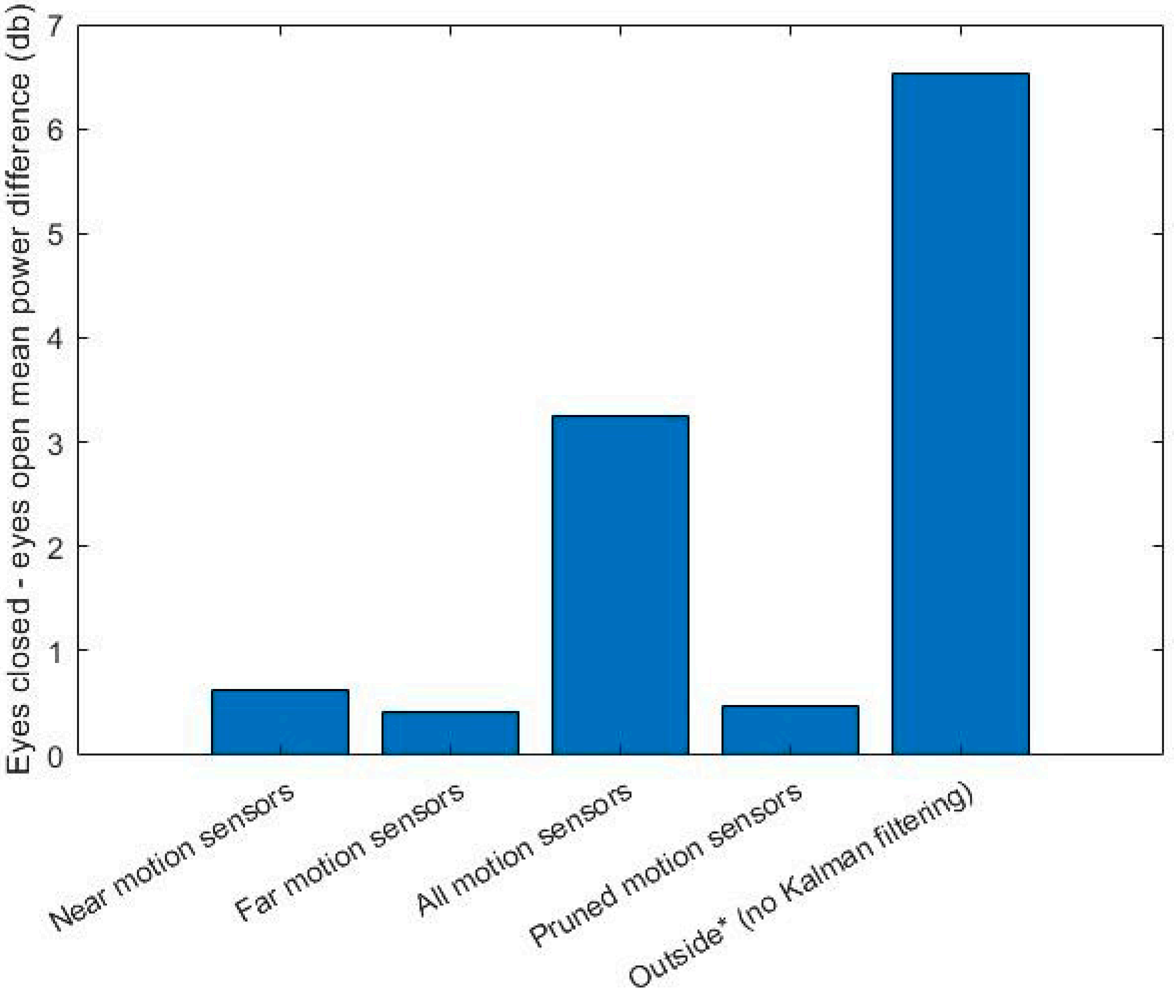

Figure S2: Mean EEG alpha power difference between eyes-open and eyes-closed states after artifact removal, using different combinations of motion sensors for Kalman filtering, as compared to clean recordings outside the MRI scanner.

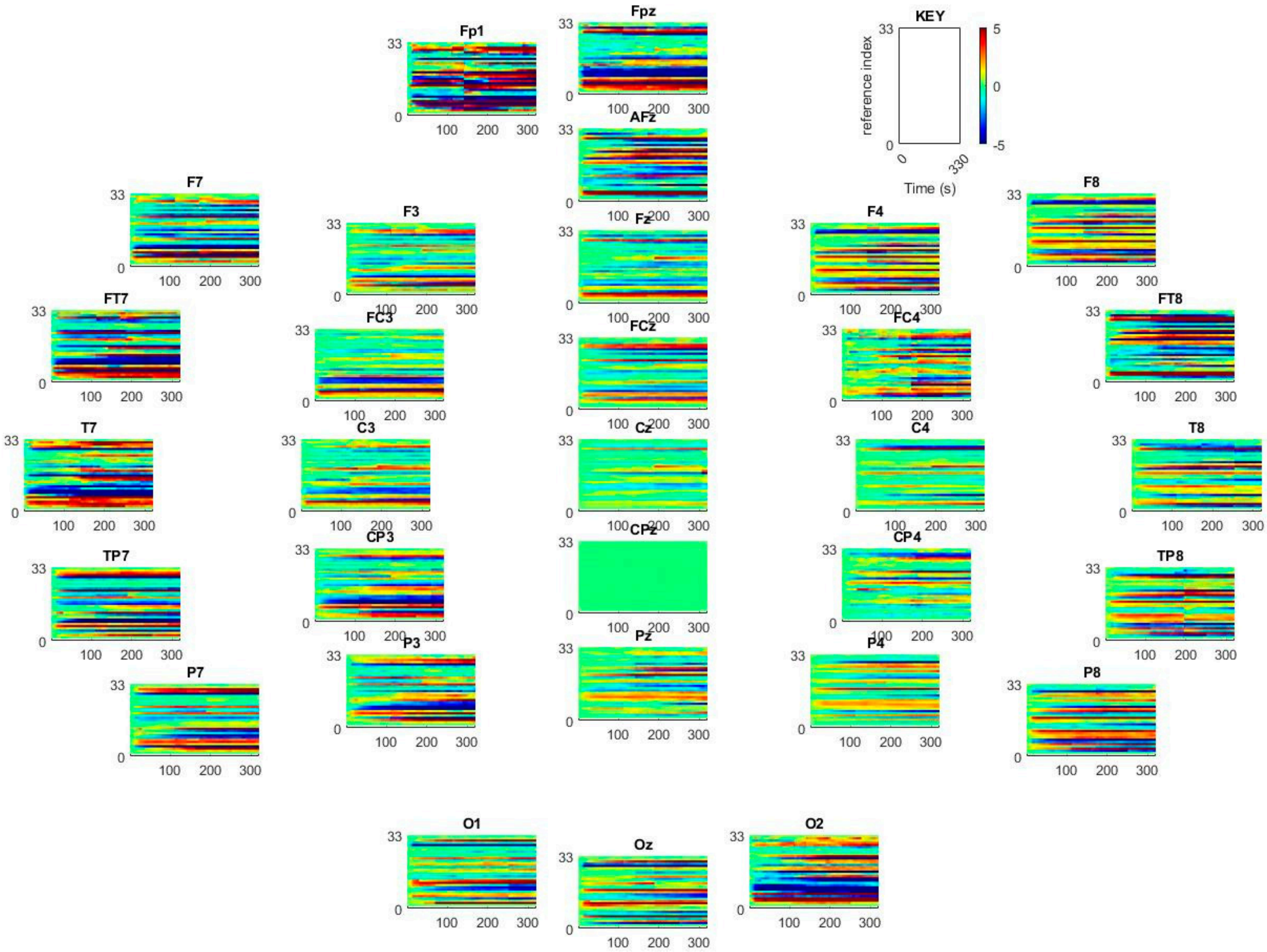

Figure S3: Example of state vectors $\mathbf{x}_{i,t}$***.*** *The plots show that the weights change over time as they appear as horizontal lines with different colors or different values. For many channels, the lines become steady over time, after the typical Kalman filter learning period.*

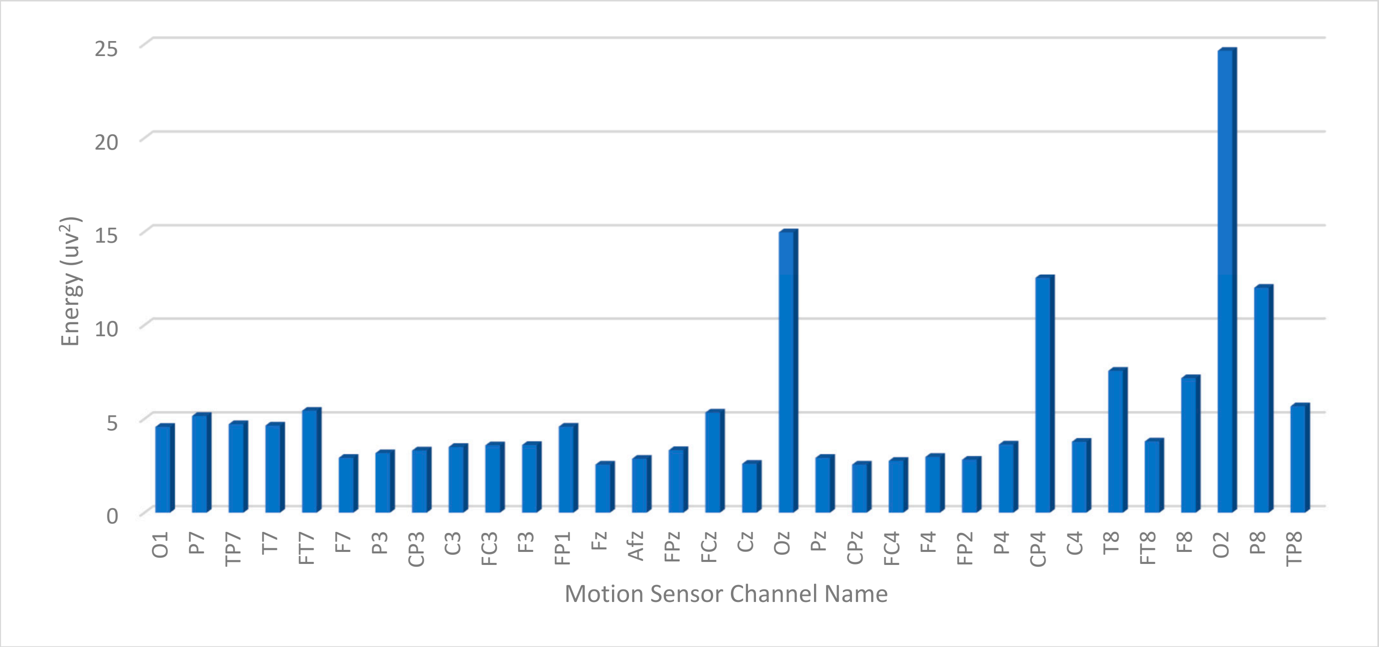

Figure S4: Example of energy in a 10 s recording of motion sensors recorded at 3 T in a volunteer with the MotoNet.

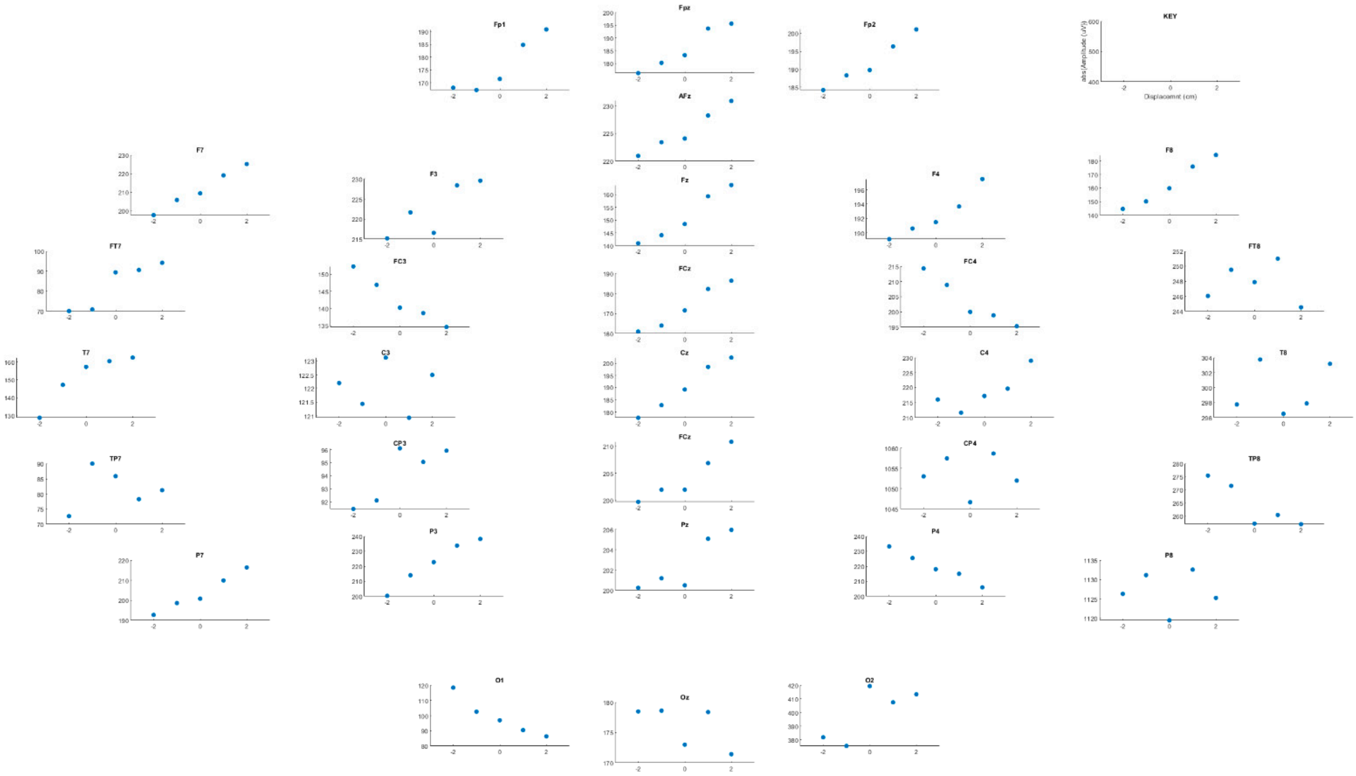

Figure S5: Channel by channel x-axis shift vs. motion sensor amplitude when running the custom sequence at 3T MRI. *Most channels exhibit signal amplitudes that are a linear function of position. The outliers may be generated by damaged traces, faulty connections, or sinusoids that are too noisy for amplitude detection, and were excluded from the average.*

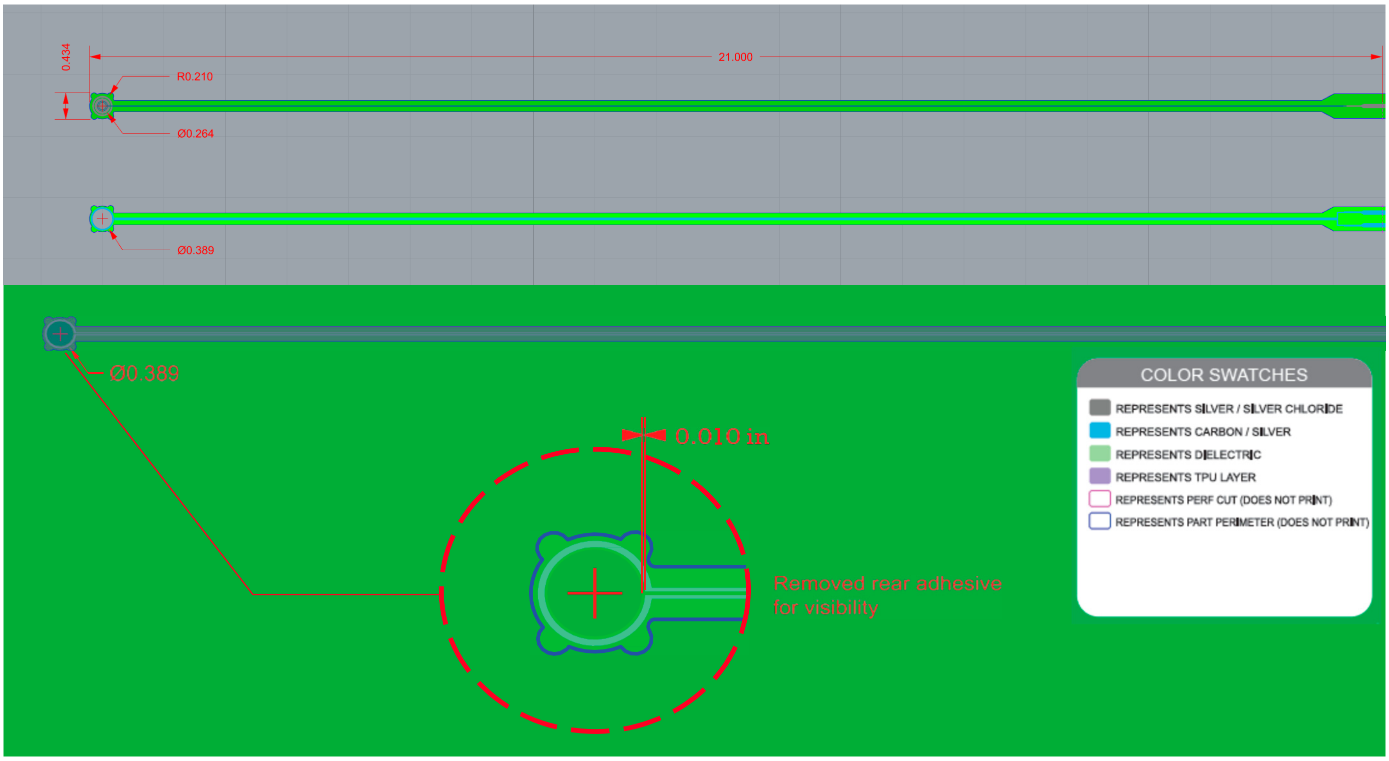

Figure S6: The MotoNet CAD design. *(Top): CAD design of the double sided PTF: one side has a traditional EEG electrode connected to a single terminal, and the other side has a PTF coil connected to two terminals. (Bottom): a zoomed-in view of the PTF coil.*

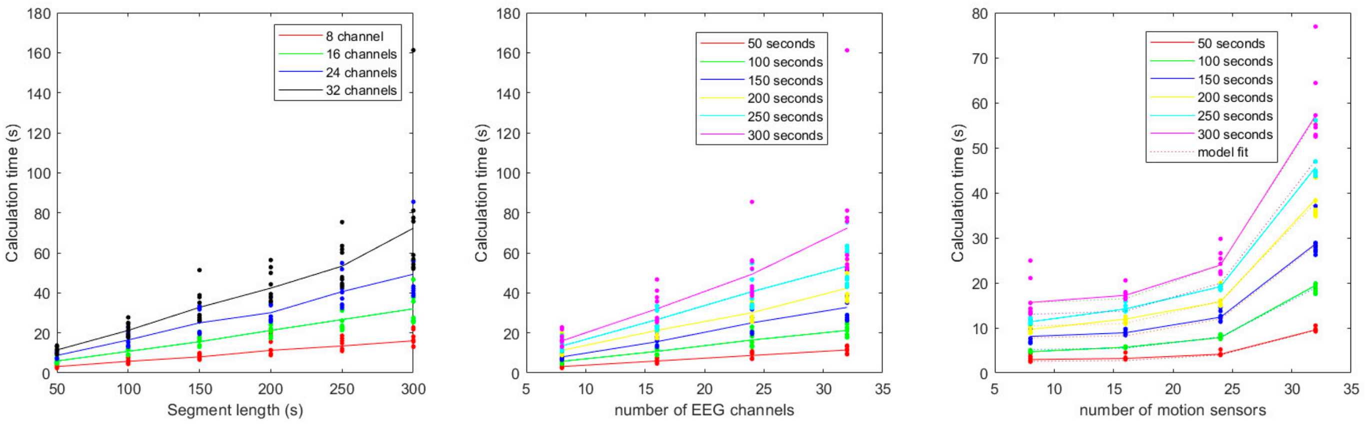

Figure S7: Computational complexity of the adaptive noise removal algorithm. *(Left) Computation time varies approximately linearly with the segment length. (Center) Computation time varies approximately linearly with number of EEG channels. (Right) computation time varies exponentially with the number of motion sensor inputs. Solid lines show mean, dotted lines show best fit with an exponent of 5.5.*

```matlab
function [Recovered_eeg_Kalman] = KalmanMotonet(EEG, K_Targets, q, R)
%   Performs Kalman filter offline to remove BCG artifact
% Inputs : EEG - data matrix, n samples x m channels (including both EEG and
%              sensor channels. Sensors assumed to columns 1-32
%          K_Targets - a vector indicating which channel numbers to filter
%          q - a scalar hyperparameter, which must be tuned for optimal
%              performance. A reasonable starting point is 1.0e-4
%          R - a scalar hyperparameter, which must be tuned for optimal
%              performance. A reasonable starting point is 10000
%
% Outputs : Recovered_eeg_Kalman - clean data matrix, n samples x
%              length(K_targets) channels



[n_all, nChans] = size(EEG); % detect dataset size
numEEG = length(K_Targets);

% motion sensors are channels 1-32
refChans = 1:32;

DM = [ones(n_all, 1), EEG(:, refChans)]'; % build our design matrix from reference channels
% include extra reference channel with a constant value.

SynthEEG = EEG(:, K_Targets)';  % select target channels

% initialize algorithm variables
j = length(refChans) + 1; % number of references + 1

x_hat = zeros(j, numEEG); % intialize x_hat to zeros
h_hat = zeros(n_all, numEEG);
Q = eye(j) * q; % initialize Q to be identity matrix times a scalar hyperparameter
P = eye(j); % initialize P to be an identiy matrix
P = repmat(P, 1, 1, numEEG);
for chan = 1:numEEG % loop through target channels

    channelDM = DM;

    for t = 1:n_all % loop through time
        % perform Kalmfilter update on a sample-by-channel basis
        x_hat_givenPrevious = x_hat(:, chan); % get x_hat from previous time step
        P_givenPrevious = P(:, :, chan) + Q;
        KalmanGain = P_givenPrevious * channelDM(:, t) ... % calculate filter gain
```

```
                * (channelDM(:, t)' * P_givenPrevious * channelDM(:, t) + R) ^-1;
        x_hat(:, chan) = x_hat_givenPrevious + ... % update x_hat for this timestep
                KalmanGain * (SynthEEG(chan, t) - channelDM(:, t)' * x_hat_givenPrevious);
        P(:, :, chan) = (eye(j) - KalmanGain * channelDM(:, t)') * P_givenPrevious; % update P for this timestep
        h_hat(t, chan) = channelDM(:, t)' * x_hat(:, chan); % model BCG signal for this timestep from sensor signals and weights
    end
end
% subtract modeled BCG from the original EEG signal
Recovered_eeg_Kalman = (SynthEEG - h_hat')';
end
```
